# Culturing and Scaling up Stem Cells of Dental Pulp Origin Using Microcarriers

**DOI:** 10.3390/polym13223951

**Published:** 2021-11-15

**Authors:** Anna Földes, Hajnalka Reider, Anita Varga, Krisztina S. Nagy, Katalin Perczel-Kovach, Katalin Kis-Petik, Pamela DenBesten, András Ballagi, Gábor Varga

**Affiliations:** 1 Department of Oral Biology, Semmelweis University, H-1089 Budapest, Hungary; foldes.anna@dent.semmelweis-univ.hu (A.F.); reider.hajni@gmail.com (H.R.); anita.varga.001@gmail.com (A.V.); nagy.krisztina@dent.semmelweis-univ.hu (K.S.N.); perczel.katalin@dent.semmelweis-univ.hu (K.P.-K.); 2 Department of Applied Biotechnology and Food Science, University of Technology and Economics, H-1089 Budapest, Hungary; aballagi@diagon.com; 3 Institute of Biophysics and Radiation Biology, Semmelweis University, H-1089 Budapest, Hungary; kispetik.katalin@med.semmelweis-univ.hu; 4 Department of Community Dentistry, Semmelweis University, H-1089 Budapest, Hungary; 5 Department of Orofacial Science, University of California, San Francisco, CA 94143, USA; Pamela.DenBesten@ucsf.edu; 6 Gedeon Richter Plc, H-1089 Budapest, Hungary; 7 Centre for Translational Medicine, Semmelweis University, H-1089 Budapest, Hungary

**Keywords:** stem cells, mesenchymal, scaffold, Cytodex 1, Cytopore 2, dental pulp, scaling up, shake flask, microcarrier

## Abstract

Ectomesenchymal stem cells derived from the dental pulp are of neural crest origin, and as such are promising sources for cell therapy and tissue engineering. For safe upscaling of these cells, microcarrier-based culturing under dynamic conditions is a promising technology. We tested the suitability of two microcarriers, non-porous Cytodex 1 and porous Cytopore 2, for culturing well characterized dental pulp stem cells (DPSCs) using a shake flask system. Human DPSCs were cultured on these microcarriers in 96-well plates, and further expanded in shake flasks for upscaling experiments. Cell viability was measured using the alamarBlue assay, while cell morphology was observed by conventional and two-photon microscopies. Glucose consumption of cells was detected by the glucose oxidase/Clark-electrode method. DPSCs adhered to and grew well on both microcarrier surfaces and were also found in the pores of the Cytopore 2. Cells grown in tissue culture plates (static, non-shaking conditions) yielded 7 × 10^5^ cells/well. In shake flasks, static preincubation promoted cell adhesion to the microcarriers. Under dynamic culture conditions (shaking) 3 × 10^7^ cells were obtained in shake flasks. The DPSCs exhausted their glucose supply from the medium by day seven even with partial batch-feeding. In conclusion, both non-porous and porous microcarriers are suitable for upscaling ectomesenchymal DPSCs under dynamic culture conditions.

## 1. Introduction

Dentally derived ectomesenchymal stem cells are promising sources for cell therapy, tissue engineering, disease modeling and drug discovery. These cells are easily accessible and hold multipotent differentiation capacity [1,2]. Since the discovery of dental pulp stem cells (DPSC) in adult teeth [3], other dental sources have also been identified, including the pulp of exfoliated deciduous teeth [4], the periodontal ligament [5], the dental follicle [6] and the apical papilla of the developing tooth root [7].

DPSCs are known to share common properties with bone marrow mesenchymal stromal cells [8], expressing CD73, CD90, CD105 and Stro-1 cell surface markers [9], mesenchymal stem cell markers such as nestin and vimentin [10,11,12], and also osteogenic markers such as osteonectin and bone sialoprotein [8,13]. Furthermore, they also show similar, but not identical, differentiation capabilities with other mesenchymal stem cells [8,13]. DPSCs, just like other dentally derived ectomesenchymal stem cell populations are derived from neuroectoderm and are able to differentiate not only into osteogenic, adipogenic, chondrogenic, and myogenic lineages, but also to a neurogenic lineage [8,10,11,14]. Therefore, DPSCs may serve not only for regenerative dentistry and oral surgery [1,15], but also for the replacement of neuronal cells [16]. To support regenerative therapies, the stem cells in dental pulp also hold immunomodulatory properties [17,18,19]. 

The wide range of available literature uses diverse definitions for the term “DPSC culture”. Gronthos et al. first defined all cells isolated from the dental pulp that are anchorage-dependent, attaching to plastic cultivation surfaces but not to glass, as dental pulp stem cells [3]. Cells in these cultures uniformly show stem cell/progenitor cell marker proteins including nestin and vimentin, but they are also somewhat heterologous, as indicated by the expression of Stro-1, which is generally regarded as a mesenchymal stem cell marker. Stro-1 is exhibited by 10–20% of the cell population, and its expression is dependent on the type of applied medium, FBS concentration and passage number [12]. These cells are clearly regarded as multipotent, but the differentiation potential does not apply only to the small portion of Stro-1 positive cells in the DPSC cultures. Previous quantitative studies by us and by others have clearly demonstrated that the majority of cells in the culture are able to differentiate into both osteogenic [10,11,12,17] and neurogenic [10,11,20,21] lineages. Obviously, many of these are not Stro-1 positive, but are nonetheless able to show multipotency for differentiation. Therefore, in the present work the term “DPSC” is used for this non-homogenous population of cells.

DPSCs differentiate with multiple passaging, and therefore a great obstacle to the use of DPSCs is the lack of standard methods for their expansion without multiple passages. Strategies for the scaling up of stem cells include: (1) monolayer cultures, (2) organoid/spheroid production, and (3) culturing on microspheres using either static or dynamic culture conditions [22]. Monolayer cultivation is the least effective method, yielding only a limited number of cells. In organoid/spheroid culture, three-dimensional cell aggregates are produced, usually under static conditions but the process is also possible under dynamic conditions. This approach is thought to mimic the three-dimensional in vivo environment [23,24]. The challenge of this strategy is to expand cells using controlled conditions without causing harmful effects on the cells or undesirable cell differentiation [25,26]. Furthermore, difficulties in spheroid manufacture include problems controlling spheroid size, cellular fate, and cell necrosis within the organoid spherical structures [24,27,28]. Therefore, the best outcome, in terms of achieving the highest yield in scaling up, seems to be the application of microcarriers combined with dynamic culture systems such as shake flasks, spinner flasks and bioreactors [22,29,30,31]. Microcarriers are principally designed for use in suspension culture systems for the growth of adherent cells. The application of microcarriers in dynamic culture systems may provide yields of tens of millions of cells [29,30,31]. 

The mesenchymal elements of the tooth, including the dental pulp, are derived from the cranial neural crest ectomesenchyme, which actually originates from the ectoderm and not the mesoderm [32,33]. These cells are similar to, but in many respects also quite different from mesoderm-derived mesenchymal tissues [32,33]. Recent pioneering studies have clearly demonstrated that MSCs from different origins exhibit very different proliferation and differentiation potentials and also diverse interactions with various scaffolds [34,35,36,37]. In our proof-of-concept study we have aimed to demonstrate, for the first time, the successful quantitative expansion of ectomesenchymal cells, in this case DPSCs. This is very important since previous studies were always performed on conventional mesoderm-derived mesenchymal stem cells such as those from bone marrow and adipose tissue.

There are only a limited number of studies that report approaches to the scaling up of DPSCs [38,39,40]; however, these studies either used only static culture conditions or demonstrated only the qualitative but not the quantitative aspects of cell expansion. These studies, though limited, indicate that DPSCs can be expanded on microspheres, but the available evidence is modest and, therefore, further characterization is needed. Thus, in this proof-of-concept work we aimed to test the efficacy of two well characterized microcarrier beads, non-porous Cytodex 1 and porous Cytopore 2 [29,30,31,41], used in a dynamic shake flask system, for growing and expanding DPSCs. We hypothesized that with this system, DPSC can be grown at large scale to produce tens of millions of cells which can then be used for characterization, differentiation, and for targeted tissue engineering.

## 2. Materials and Methods

### 2.1. Cell Isolation for DPSC Cultures and Cultivation Procedures

Normal impacted third molars were surgically removed from healthy young adults (18–40 years of age) at the Department of Oral Diagnostics, Semmelweis University, Budapest. Surgeries were approved by the Semmelweis University Regional and Institutional Committee of Science and Research Ethics (17458/2012/EKU and 25459/2019/EKU). DPSCs were isolated as previously described [10,11,12]. Briefly, after removing the periodontal tissue, teeth were cleaved into two with a dental bur and pulp tissue was pulled out from the pulp chamber and root canal with the use of a sterile needle. The pulp tissue was then minced and digested in 1 mL collagenase type I (1 mg/mL) (Sigma-Aldrich, St. Louis, MO, USA) solution in a phosphate-buffered saline (PBS) for 1 h at 37 °C while vortexing every 10 min. After centrifugation, the cells were resuspended in α-modified Eagle Minimum Essential Medium (αMEM) (Lonza, Basel, Switzerland), supplemented with 10% fetal bovine serum (FBS, Gibco, Grand Island, NY, USA), 2 mM L-glutamine (Sigma-Aldrich, St. Louis, MO, USA), 100 units/mL penicillin and 100 µg/mL streptomycin (Gibco, Grand Island, NY, USA) in T75 tissue culture flasks (Orange Scientific, Braine-l’Alleud, Belgium) for expansion under standard conditions (37 °C, 100% humidity, 5% CO_2_). After the first passage, a partial culture medium change was completed at day 3, then subconfluent cultures were subcultivated once a week at a ratio of 1:20 with 0.25% trypsin EDTA (Gibco, Grand Island, NY, USA).

### 2.2. Morphological Observations on DPSCs Cell Cultured on Plastic Culture Dishes

Cell morphology of the DPSCs cultivated on a plastic surface was imaged by phase contrast microscopy (Nikon Eclipse TS100, Europe BV, Amsterdam, Netherlands and Nikon TMS Europe BV, Amsterdam, The Netherlands). Photomicrographs were taken and processed using Axiovision 482 (Carl Zeiss, Oberkochen, Germany)and Scion Image (Scion Corp, Frederick, MD, USA) acquisition and image analysis software. 

### 2.3. Immunocytochemical Characterization of DPSCs

Cells from third passage DPSC cultures were plated on 8-well chamber slides and, after 2 or 3 days, fixed with 4% paraformaldehyde in PBS. Triton X was used to permeabilise the cell membrane, and a 5% goat serum in PBS was used to block the binding of non-specific proteins. Anti-nestin (rabbit IgG) antibody was purchased from Sigma-Aldrich, anti-vimentin (mouse IgG) and anti-CD90/Thy-1 (mouse IgG) antibodies from Calbiochem (Calbiochem La Jolla, CA, USA), while anti-c-kit/CD117 (rabbit IgG) was purchased from Santa Cruz Biotechnology. Anti-STRO-1 antibody (mouse IgM) was generously provided by Prof. Richard Oreffo (University of Southampton, UK), while antibodies against osteonectin (ON) (LF-37, rabbit IgG), and bone sialoprotein (BSP) (LFMb-25, mouse IgG) were kind gifts from Dr. Larry W. Fisher (NIH, Stapleton, NY, USA). β-tubulin III (mouse IgG) and anti-GFAP (mouse IgG) were used to examine neurogenic differentiation. The samples were incubated overnight at 4 °C with primary antibodies diluted in PBS containing 2.5% goat serum. Next day, the samples were incubated for one hour at room temperature with Alexa Fluor 488-conjugated goat anti-rabbit IgG, anti-mouse IgG and IgM secondary antibodies (Molecular Probes, Fisher scientific, Waltham, MA, USA) diluted in 2.5% goat serum. Finally, the slides were mounted using ProLong Gold antifade reagent with DAPI (Molecular Probes, Fisher scientific, USA), which also stained the nuclei. The slides were stored in the dark at 4 °C until investigation under a fluorescence microscope (Nikon Eclipse E600, Europe BV, Amsterdam, The Netherlands). A Retiga 2000R digital CCD camera (QImaging Digital Imaging Systems Ltd., Buckinghamshire, UK)) and Image Pro software (Media Cybernetics, Rockville, MD, USA ) were used to obtain the fluorescent images.

### 2.4. Characterization of Osteogenic Differentiation of DPSCs

For the osteogenic differentiation, 104 cells/cm^2^ (from the third passage) were plated into culture dishes in the culture medium. Differentiation was induced 24 h after cell seeding using the culture medium described by Kemoun et al. [42]. During the experiments, which lasted 21 days, the osteogenic and control media were changed twice weekly without passaging. The composition of the control medium was αMEM supplemented with 1% FBS, 2 mM L-glutamine, 100 units/mL penicillin and 100 mg/mL streptomycin. The osteogenic differentiation medium was prepared from the control medium by supplementation with 50 μg/mL L-ascorbic acid 2-phosphate (BioChemica, Billingham, UK), 10 mM β-glycerophosphate (BioChemica, Billingham, UK) and 10 μM dexamethasone (Sigma-Aldrich, St. Louis, MO, USA). 

In order to visualize the calcium deposits during osteogenic differentiation, von Kossa staining was carried out. After fixation with 70% ethanol (Reanal, Budapest, Hungary), the differentiated DPSCs were treated with 5% silver nitrate under bright light. Then 5% sodium thiosulfate (Sigma-Aldrich, St. Louis, MO, USA) was used to fix the colour change. Images were acquired using an inverted phase contrast microscope (Nikon Eclipse TS100, Europe BV, Amsterdam, Netherlands), a high-performance CCD camera (COHU) and Scion Image software.

### 2.5. Characterization of Neurogenic Differentiation of DPSCs

For neurogenic differentiation, 10^3^ cells/cm^2^ from the third passage were seeded in poly-L-ornithine-coated 6-well culture dishes in culture medium. Differentiation was induced 24 h after cell seeding using the protocol described by Fugii and coinvestigators [43]. Cells were cultured for 72 h in a serum-free MEM medium supplemented with 2 mM L-glutamine, 100 units/mL penicillin and 100 mg/mL streptomycin, 20 ng/mL basic fibroblast growth factor (b-FGF) (Sigma-Aldrich, St. Louis, MO, USA) and 20 ng/mL epidermal growth factor (EGF) (Sigma-Aldrich, St. Louis, MO, USA). The medium was changed to a basic induction medium (BIM) consisting of a neurobasal medium supplemented with B27, 1 mM dibutyrylcyclic AMP (db-cAMP) (Sigma-Aldrich, St. Louis, MO, USA), 0.5 mM 3-isobutyl-1-methylxanthine (IBMX) (Sigma-Aldrich, St. Louis, MO, USA) and 200 μM ascorbic acid, cultured for 3 days and then the following factors were added to the BIM (all from Thermo Fisher Scientific, Waltham, MA, USA): 40 ng/mL BDNF 10 ng/mL GDNF, 2 ng/mL transforming growth factor β (TGF- β), and 50 ng/mL fibroblast growth factor 8 (FGF-8). Cells were incubated using this neurogenic cocktail until the completion of the experiments. They were then investigated by phase contrast microscopy and processed for immunocytochemistry as described above.

### 2.6. DPSC Culture on Microcarriers under Static Conditions

DPSCs were cultured on non-porous Cytodex 1 and porous Cytopore 2 microcarriers in 96-well plates and later in shake flasks during the scaling-up experiments. The cells were first cultivated in small volumes, using static conditions, and were also grown for microsphere experiments. For the microsphere experiments, 96-well U-bottom, non-attaching plates (Nunc, Thermo Fisher Scientific, Waltham, MA, USA) were used to prevent the attachment of the DPSCs, which adhere to the normal plastic surfaces used for cell culturing. The plates were pretreated with 2-methacryloyloxiethyl phosphoryl-choline to further prevent cell attachment. 

We used two types of microcarriers, the first was the non-porous Cytodex 1 (GE Healthcare, Chicago, IL, USA). This spherical microcarrier has positive N,N-diethylaminoethyl (DEAE) surface charges on a cross-linked dextran network with 190 µm diameter units, and 1.03 g/mL density, slightly more than the density of water, to promote sedimentation. A number of previous studies have demonstrated that this carrier is suitable as a cell-attaching carrier [29,30,31]. The second carrier was the porous Cytopore 2 (GE Healthcare, Chicago, IL, USA). This microcarrier has a sponge-like structure that enables cells to penetrate deep into the inner parts of the carrier, thereby providing a larger attachment surface, and also potentially decreasing the shear force when agitating the cells. Its basic matrix is cellulose that also has positive DEAE charges on the surfaces. The Cytopore 2 microcarrier is also spherical with an average diameter of 230 µm, and its density is similar to Cytodex 1. Cytopore 2 has also been shown to serve as a suitable carrier for cell expansion [29,30,31]. Neither Cytodex 1 nor Cytopore 2 was designed to be used for in vitro cell culture experiments. Rather, these microcarriers were constructed to perform basic characterization of various surface-attaching cell types. The Cytodex 1 matrix is biologically inert and provides a strong but non-rigid substrate for stirred microcarrier cultures. The matrix of the Cytopore 2 is also non-toxic to the cells and not biodegradable. Therefore, these microcarriers are less suitable for tissue engineering or direct transplantation because they cannot be broken down by the human body [44]. 

Before use, the microcarriers were prepared according to the manufacturer’s protocols. Briefly, 50 mg microcarriers/mL PBS were first incubated for 3 h at room temperature. Then the supernatant was decanted and the microcarriers were washed in PBS and sterilized in an autoclave (121 °C, 16 min, 1.2 Bar). They were then washed in PBS, centrifuged, and diluted in a fresh culture medium. 

### 2.7. Morphological Observations on DPSCs Cell on Microcarriers

Cell morphology of the DPSCs grown on microcarriers was studied again by phase contrast microscopy as described above. Photomicrographs were taken and processed using Axiovision 482 and Scion Image acquisition and image analysis software. 

The DPSCs on the microcarriers were also visualized by fluorescence and two-photon microscopy (Femto2D-Inverted, Femtonics, Budapest) using DAPI (Thermo Fisher Scientific, Waltham, MA, USA) and Vibrant DiD (Thermo Fisher Scientific, Waltham, MA, USA) staining, respectively. The presence of cells attached to the microcarrier surfaces was directly demonstrated by nuclear diamidino-2-phenylindole (DAPI) staining on a fluorescent microscope (Nikon TE600, Europe BV, Amsterdam, The Netherlands). These experiments were performed in 96-well plates, with each well containing 20,000 cells and 0.4 mg microcarrier in 200 µL culture medium. Samples were washed in PBS twice then fixed in 200 µL 70% ethanol for 15 min at room temperature. Afterwards, they were washed three time in 200 µL PBS for 5 min. Following that, cells were incubated with 50 µL 1 µL/mL DAPI at room temperature for 30 min on a tilting table. Finally, the samples were washed twice with PBS. Photomicrographs were taken using a Retiga 2000 cooled CCD camera and QCapture software.

We also applied a vital staining method using the Vybrant DiD reagent (Thermo Fisher) to visualize the living cells. Cells were plated in 96-well plates, into each well 100 µL volume was given, followed by a 20 min incubation at 37 °C. Afterwards, the medium was changed and the cells washed again three times. After 24 h incubation the cells were fixed in 200 µL 4% paraformaldehyde (PFA) (Sigma-Aldrich, St. Louis, MO, USA) at room temperature for two hours on a tilting table. The samples were then washed in PBS twice. Images of the Vybrant DiD-stained cells on microspheres were taken with a two-photon microscope (Femto2D-Inverted, Femtonics, Budapest) equipped with Olympus objectives UPLSAPO10X2 and LUMPLFLN40XW. The excitation wavelength was 780 nm, and emission was detected in two channels: autofluorescence of the carrier in the green channel (490–560 nm) and fluorescence of the cells in the red channel (600–700 nm). 

### 2.8. Cell Viability Evaluation

Cell viability was evaluated using the alamarBlue assay (Thermo Scientific, Waltham, MA, USA) according to the manufacturer’s protocols. This assay is based on a resazurin reduction to resorufin, a compound that is red in color and highly fluorescent in living cells. Thus, the living cell number is proportional to the chemical reduction of non-fluorescent resazurin to fluorescent resorufin. Cells were plated in 96-well plates, and experiments started 24 h after plating. The determination of the changes in cell number was completed between days 1 and 14 of culture following incubation with the dye under standard conditions. AlamarBlue fluorescence was detected at 590 nm (with excitation at 560 nm) using a Perkin-Elmer LS50B luminescence spectrometer (PerkinElmer, Inc., Waltham, MA, USA). Results are expressed as the number of cells in a given well or flask. 

The cell viability test was optimized for DPSCs growing both on plastic and on microcarriers, so that the measured values fell into the linear part of the calibration curve. In both cases the cells were pre-cultivated in flat-bottom 96-well plates. The resazurin-containing reaction solution was applied to the cells at a 1:10 ratio, that is 25 µL reagent was added to the 250 µL culture volume. After 4 h incubation in the plates not containing microcarriers, measurements could be made directly. In the case of the microcarriers, 100 µL supernatant was transferred to a new 96-well plate for the spectrophotometric measurements. 

For static cultivation of the DPSCs without microcarriers, cells were grown in conventional 96-well plastic plates and cultivated for 1, 4, 7 and 14 days. Cells were seeded at 7500 or 22,500 cells/well initial densities. At the end of the cultivation period we followed the process described above to obtain the actual number of cells. For the static microcarrier experiments, we followed a similar procedure after preparing cell-microsphere suspensions as described above. 

### 2.9. Scaling-up Experiments under Dynamic Conditions 

In these experiments DPSCs from passage 3–6 were first grown in T75 tissue culture flasks. The cell number was determined in a Burker chamber and the cells were prepared as a 10^6^ cell/mL stock solution in culture medium. Microcarriers were also prepared as described above and diluted in the culture medium to a 12 mg carrier/mL stock concentration. Each glass shake flask had 125 mL volume (Corning, Glandale, CA, USA) as DPSC cells do not attach to glass surfaces. The 125 mL flasks were first filled with 35.7 mL culture medium. Then 7.5 mL Cytodex 1 or Cytopore 2 microcarrier-containing stock solution was added. Five ml of the DPSC stock solution was pipetted into each flask. Finally, the flasks were gently shaken by hand to give an initially homogenous incubation suspension. The final concentration of the mixture was 1.8 mg/mL for the microcarriers and 10^5^ cell/mL for the DPSCs; in each flask the starting cell number was 5 × 10^6^ stem cells. Half of the flasks were placed on a Thermo Max 2000 shaker, standing within a CO_2_ incubator at 37 °C temperature and 5% CO_2_ concentration, and running at 100 rpm. The other half of the flasks stood still for 12 h within the incubator before the shaking started. This way we formed four treatment groups: CD1 for Cytodex 1 shaken; CD1-ON for Cytodex 1 overnight unshaken then shaken; CP2 for Cytopore 2 shaken; and CP2-ON for Cytopore 2 overnight unshaken then shaken.

Following the overnight procedures described above, all flasks were shaken in the incubator at 100 rpm and processed in exactly the same manner. During cultivation, the medium was partially replaced at days 2 and 4. After two days of incubation, batch-feeding was performed by adding 25 mL fresh medium to each flask. Then on the fourth day the shaking of the flasks was briefly stopped to allow for the sedimentation of the microspheres. 25 mL supernatant was pipetted out and replaced with 25 mL pre-warmed, fresh culture medium. On the seventh day the incubation was completed. Samples were collected on days 1, 2, 4 and 7. For the glucose consumption measurements 0.5 mL supernatant was taken. For the cellular investigations a 1.5 mL suspension was taken containing cell-loaded microcarriers. This experimental arrangement served as our dynamic DPSC cultivation system. 

### 2.10. Glucose Consumption of DPSCs 

Glucose consumption in the cultures was monitored by using a glucose oxidase/Clark electrode-based method (ANALOX DiaGM8, Analox Instruments Ltd., Amblecote, Stourbridge, UK) utilizing the production of D-gluconic acid from D-glucose by the glucose oxidase enzyme. Measurements were performed in three parallels using a calibration curve. Glucose consumption was estimated in DPSCs grown on microcarriers in both static and dynamic cultivation conditions. For static experiments, cells were cultured in non-attaching 96-well plates for 1, 4 and 7 days. 20,000 cells/well were cultured in 200 µL medium and 0.2 mg microcarrier. For the enzymatic reaction 100 µL supernatant was used. In the shake flask cultures, samples were taken from the supernatant of the flask as described above. 

### 2.11. Statistics 

Data are expressed as arithmetic means ± standard errors of the mean ± SEM. In each experiment at least five biological parallels were used originating from the DPSCs from the wisdom tooth of five different patients. The statistical assessment of data was performed in Prism (GraphPad) software using ANOVA followed by Bonferroni tests. A difference was considered statistically significant if * *p* < 0.05, ** *p* < 0.005, *** *p* < 0.001.

## 3. Results

### 3.1. Morphological Characterization of DPSCs

DPSCs isolated from impacted wisdom teeth adhered to the surface of plastic culture dishes within a few hours after seeding and began to divide under standard culture conditions. The vast majority of the cultured cells were fibroblast-like and spindle-shaped, similar to the reports of others [3,11,12,45] and ourselves [11,12]. After isolation, they soon adhered to the culture dishes. During the first medium change, we also removed most of the cell debris and floating non-attached cells. The cells formed a subconfluent monolayer in a T75 culture flask at week two after isolation, and a confluent monolayer at week three; however, their division slowed down due to contact inhibition between cells at this stage so that passaging was necessary. The doubling time of the cell population was about two days, and the cultures were passaged when 70–80% confluency was reached, usually once or twice a week. The DPSC cultures established in this way sustained growth for at least 15 passages. Experiments in the present study used passage numbers 3–6.

**Figure 1:**
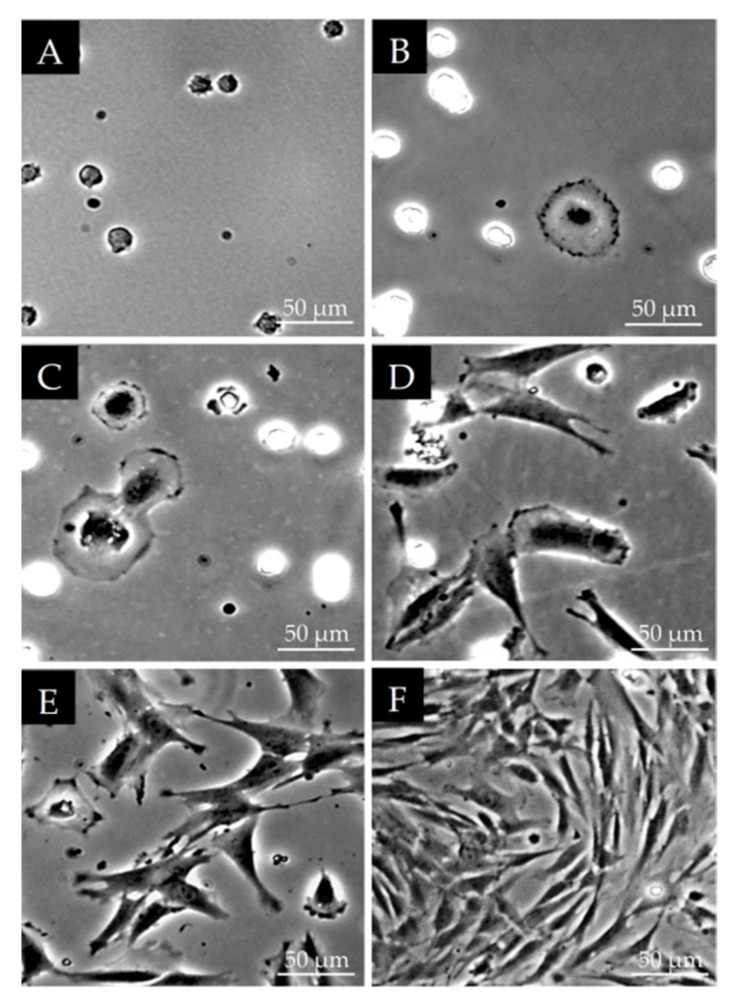
Changes in DPSC morphology at different stages of cell adhesion after passaging with trypsin/EDTA: 5 min (**A**), 1 h (**B**), 2 h (**C**) 8 h (**D**) 21 h (**E**) and 3 days (**F**). all of the photomicrographs were taken at the same magnification under a phase contrast microscope. The scale bars indicate 50 µm.

For the sub-cultivation of the cells, a trypsin/EDTA solution was used to obtain a cell suspension. After seeding these cells, we examined the morphological changes during cell adhesion. The cells formed groups with a surface-adherent, clonogenic, fibroblast morphology, similar to those described for mesenchymal stem cells of bone marrow origin. After 5 min of seeding, most of the cells in the suspension were still rounded (Figure 1A). After 1 h, some cells were already attached to the surface of the culture dish and temporarily spread as large round areas (Figure 1B). At 2 h, we could already see dividing cells (Figure 1C). At 8 h, all of the living cells clearly adhered to the bottom of the culture dish and showed a fibroblast-like morphology (Figure 1D). At 21 h (Figure 1E) and 3 days after passaging (Figure 1F) a typical fibroblast-like morphology was seen as the cell density grew with time.

### 3.2. Stem Cell Marker Characteristics of DPSCs 

**Figure 2:**
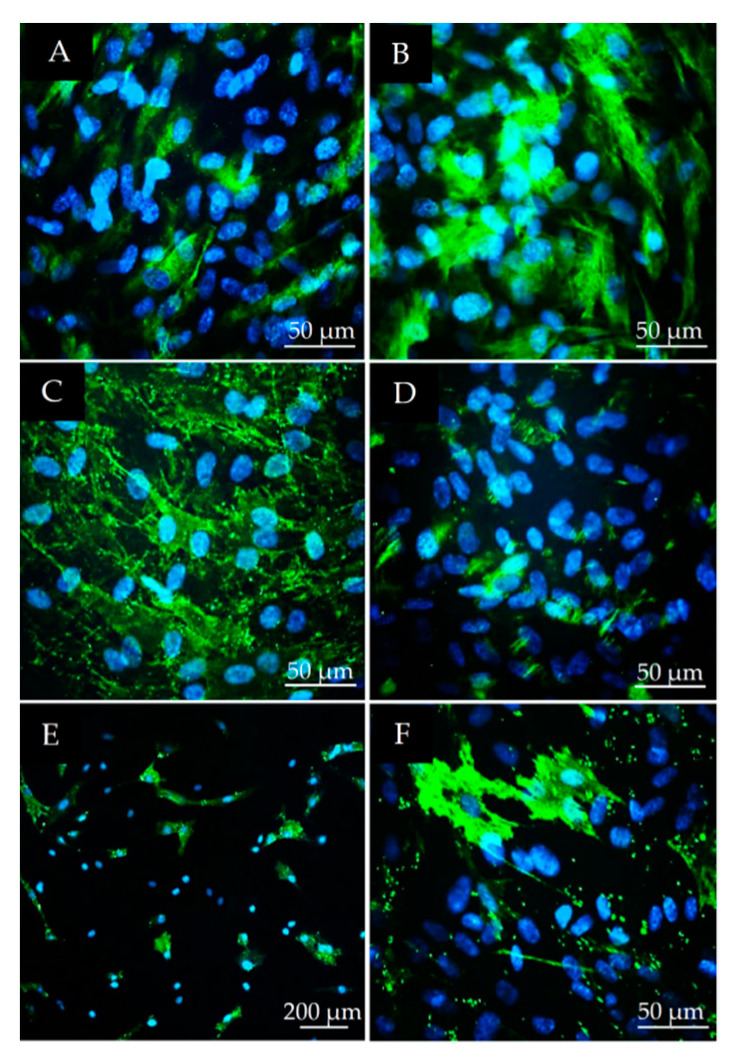
Immunofluorescent detection of the following mesenchymal and stem cell markers in DPSC cultures (passage 3): nestin (**A**), vimentin (**B**), CD90 (**C**), c-kit/CD117 (**D**), STRO-1 (**E**,**F**). The scale bars indicate 50 µm (**A**–**D**,**F**) and 200 µm (**E**). Green fluorescence shows specific immunostaining (Alexa Fluor 488) while blue shows the cell nuclei (DAPI).

Among the mesenchymal stem cell markers tested, nestin was expressed by a small proportion of the cells (Figure 2A), while vimentin (Figure 2B) and CD90 (Figure 2C) were expressed by almost all cells in the DPSC cultures. The c-kit marker was detected only in a small subpopulation of cells in these undifferentiated cell cultures (Figure 2D). The STRO-1 marker was detected in approximately 15% of the whole population (Figure 2E,F). The morphology of STRO-1 positive cells frequently differed from the usual shape of fibroblast-like dental stem cells. STRO-1 positive cells were often flattened and spread over very large areas and had more cell processes than most cells in the culture (Figure 2E,F).

### 3.3. Osteogenic and Neurogenic Differentiation Potential of DPSCs 

**Figure 3:**
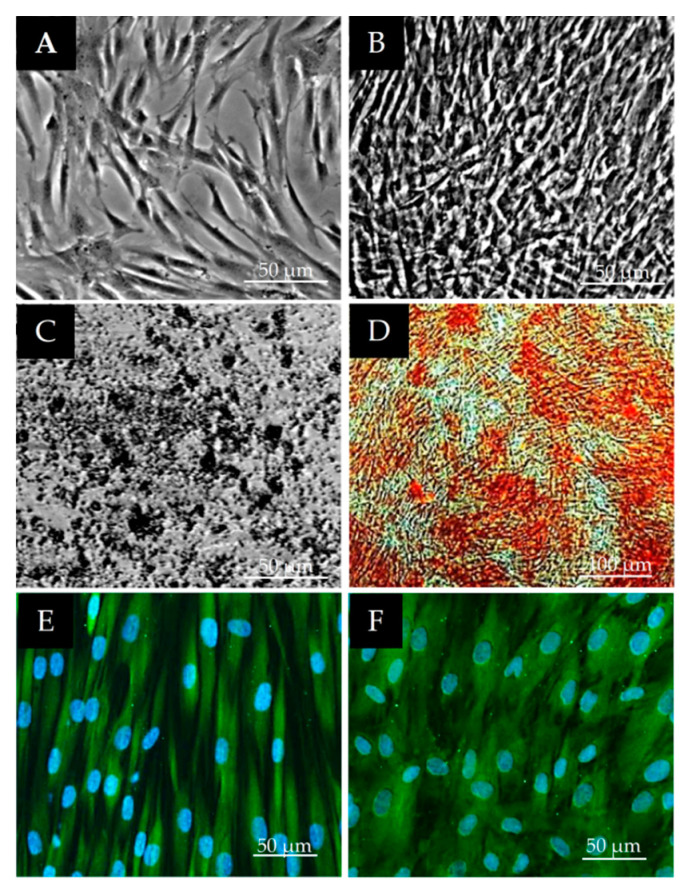
Differentiation analysis of DPSCs in osteogenic direction. (**A**–**C**) phase contrast microscopic images on day 0 (**A**) and on day 21 of osteogenic differentiation in control (**B**) and osteogenic (**C**) groups. (**D**) day 21 osteogenic group with von Kossa staining. (**E**,**F**) immunofluorescent detection of osteogenic markers on day 21 of osteogenic differentiation: osteonectin–ON (**E**) and bone sialoprotein–BSP (**F**). The bars indicate 50 µm or 100 µm. Green fluorescence shows specific immunostaining (Alexa Fluor 488) while blue shows the cell nuclei (DAPI).

The changes in morphology and density of cell cultures during osteogenic differentiation were examined first by phase contrast microscopy. The calcium deposits at the mineralizing stage of differentiation were visualized by von Kossa staining. On day 7, cell cultures of both control and osteogenic groups reached confluence but extracellular calcium was not yet detected. By day 14, a larger amount of extracellular calcium was stained, which increased significantly by day 21. No signs of mineralization were detected in the control group at any time point investigated (data not shown). The overwhelming majority of the differentiated DPSCs expressed osteonectin (ON) and bone sialoprotein (BSP) (Figure 3).

**Figure 4:**
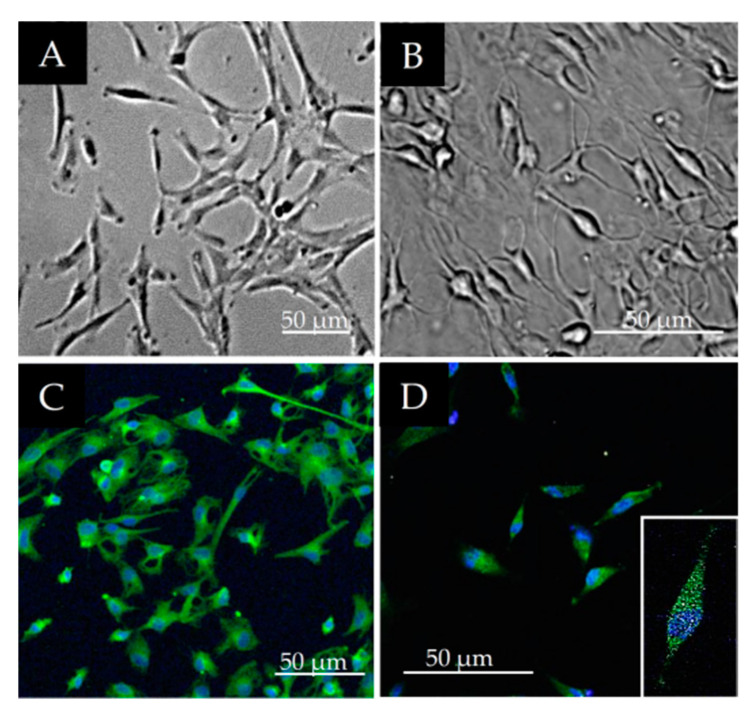
(**A**,**B**) morphological changes during neuronal differentiation of DPSCs. Phase contrast microscopic images on day 0 (**A**) and on day 12 of neurogenic differentiation (**B**). (**C**,**D**) expression of neuronal/glia markers in DPSCs after 12 days of differentiation: β-tubulin III (**C**) and glial GFAP intermediate filament protein (**D**).

The changes in cell morphology and density of the DPSC cultures during neurogenic differentiation were examined first by phase contrast microscopy. Before differentiation the DPSC cells had a spindle-shaped morphology as typical of MSC cells. (Figure 4A). During the earlier stage of differentiation their morphology reverted to a round cell shape and at the same time the cells’ body size became halved (data not shown). After 12 days of neurogenic differentiation, the vast majority of the cells displayed complex neuronal morphology, exhibiting bipolar and stellate forms (Figure 4B). Immunocytochemical analysis was performed on the 12th day of differentiation. Both the neuron-specific microtubule marker N-tubulin (Figure 4C) and glia intermediate filament marker GFAP were observed in the differentiated cells (Figure 4D). 

### 3.4. Morphological Observations on DPSCs Cultivated on Microcarriers 

**Figure 5:**
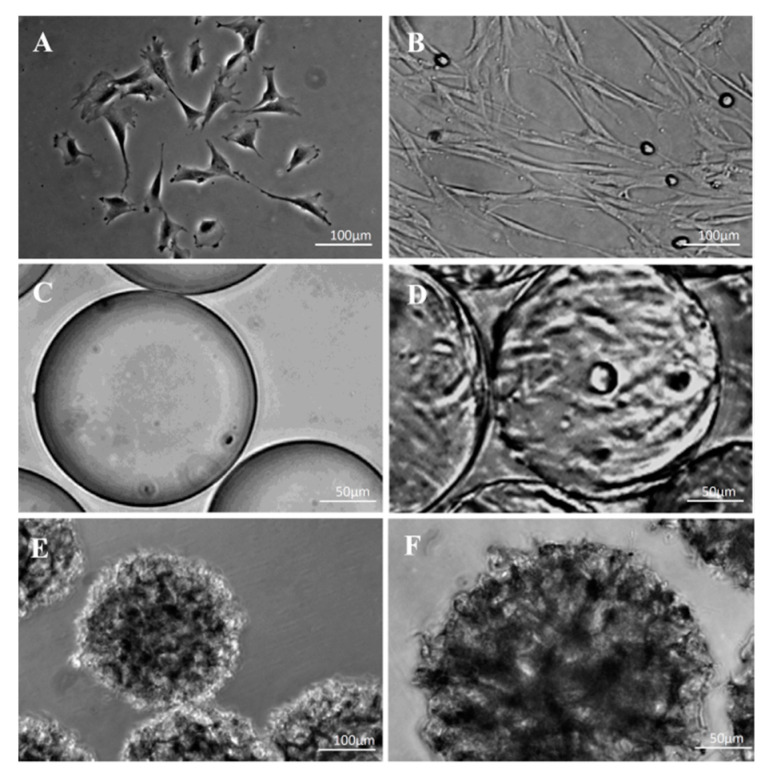
Phase contrast microscopy images of DPSCs grown on plastic surface and on microcarriers. (**A**,**B**) morphology of cultivated DPSCs on plastic surface under static conditions without microcarriers. (**C**) non-porous Cytodex 1 microcarriers. (**D**) attachment of DPSCs to the non-porous Cytodex 1 microcarriers. (**E**,**F**) cell attachment is not visible on Cytopore 2 because of the rough surface of this porous microcarrier. The scale bar indicates 100 µm (**A**–**C**,**E**) or 50 µm (**D**,**F**).

DPSCs cultivated in special, non-attaching wells in the presence of Cytodex 1 adhered to the microcarrier. In these non-attaching plates, the DPSCs were not viable until they attached to the microcarriers. The cell shape on Cytodex 1 showed an elongated spindle-like morphology (Figure 5C,D). The cells were not visible by phase contrast microscopy on the Cytopore 2 microcarriers because of their rough surface structure (Figure 5E,F). 

**Figure 6:**
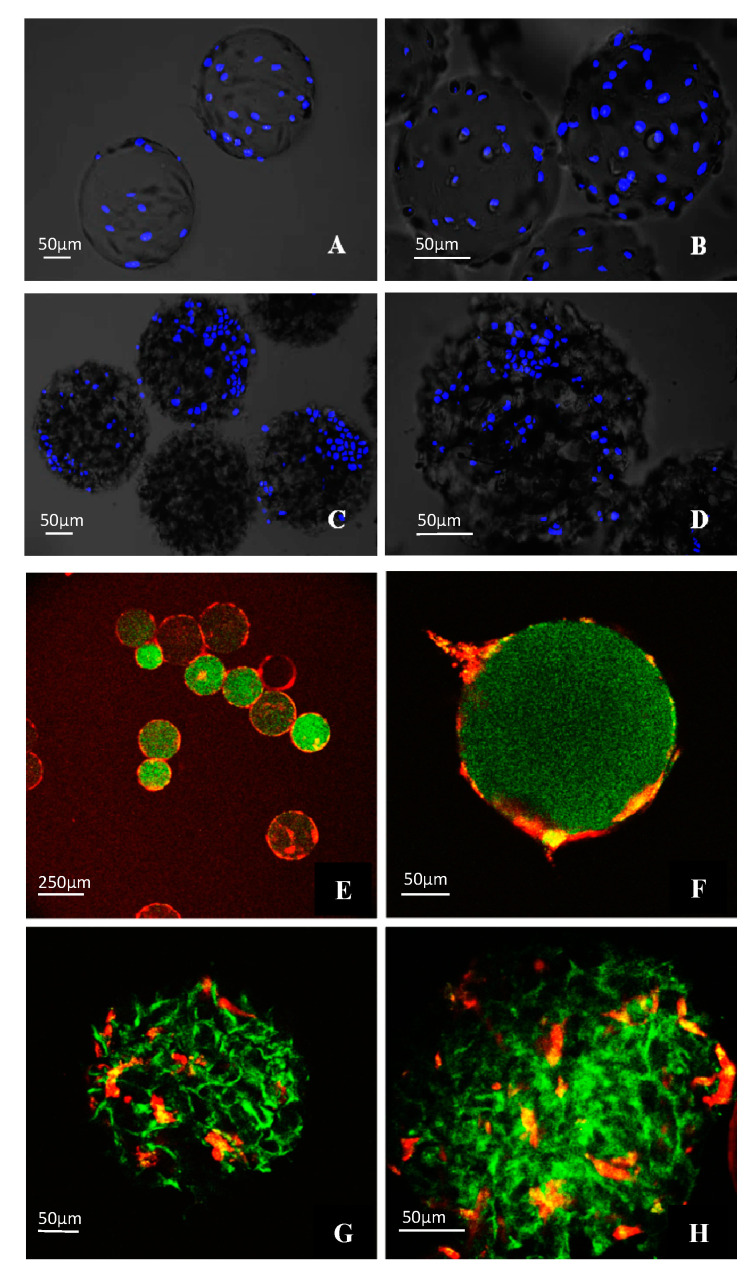
Fluorescence microscopy (**A**–**D**) and two-photon microscopy images (**E**–**H**) of DPSCs grown on microcarriers. (**A**–**D**): cell nuclei visualized by fluorescence microscopy after DAPI staining. (**E**–**H**): Vybrant DiD pre-stained cells on the microspheres using two-photon microscopy. (**A**,**B**,**E**,**F**): Cytodex 1. (**C**,**D**,**G**,**H**): Cytopore 2. The scale bar indicates 50 µm or 250 µm (**E**).

To clearly demonstrate the presence of DPSCs on the microcarriers, a fluorescent dye, DAPI, that selectively binds to DNA, was used. Figure 6A–D clearly shows that the DPSCs are bound into the surface of both the Cytodex 1 and Cytopore 2 in fluorescence microscopy. The DPSCs were distributed evenly on the surface of Cytodex 1 (Figure 6A,B), while their distribution on Cytopore 2 was uneven, and probably determined by local disunity of the porous microcarrier (Figure 6C,D).

To investigate whether the attached cells were able to enter the inner space of the Cytopore 2 microcarriers, two-photon fluorescence microscopy was applied using DPSCs pre-stained with Vybrant DiD vital dye (Figure 6E–H). As expected, the cells remained on the surface of the Cytodex 1 (Figure 6E,F) as they were unable to enter into the stable cross-linked dextran structure. On the other hand, the DPSCs were able to enter into the pores of the Cytopore 2 (Figure 6G,H). In this arrangement the cells may be better protected against shear stress, which could be an advantage during long term cultivation in a bioreactor or in other continuously shaking culture conditions. A three-dimensional picture, demonstrating that DPSCs loaded on Cytopore 2 enter into the pores of the microcarrier forming a complex structure, was taken also by two-photon fluorescent microscopy, and is shown in a video in Supplementary Figure S1.

### 3.5. Viability of DPSCs Cultured under Static Conditions

**Figure 7:**
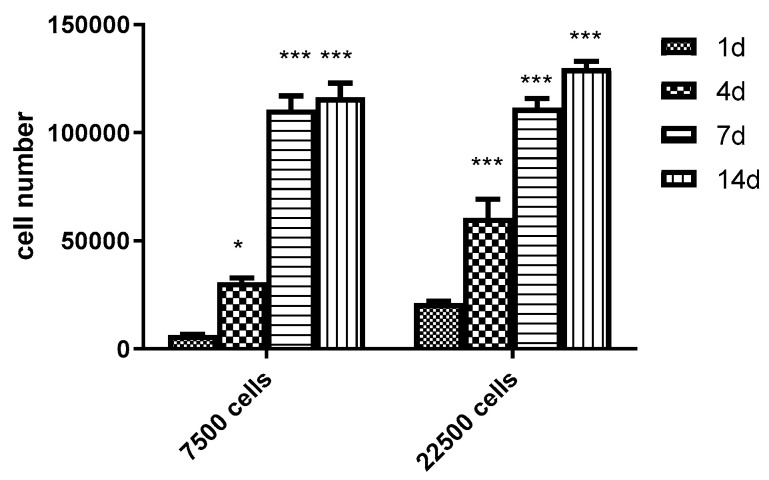
Changes in the number of viable DPSCs cultured in 96-well conventional culture plates under static conditions for 14 days. Initially 7500 and 22,500 DPSCs were seeded into the wells. Values are means ± SEM (*n* = 5). * *p* < 0.05, *** *p* < 0.001 compared with day 1.

During small scale, static cultivation, DPSC cells were first cultivated for up to 14 days without microspheres in conventional 96-well plastic plates. In these experiments 7500 and 22,500 cells/well densities were used. Cell growth was continuous up to day 7, reaching a plateau at that time. No further substantial increase in cell numbers was seen, most probably because of the contact inhibition at this cell density. At days 7 and 14 there was no difference in the viable cell mass between culture plates initially having 7500 or 22,500 cells/well (Figure 7). 

**Figure 8:**
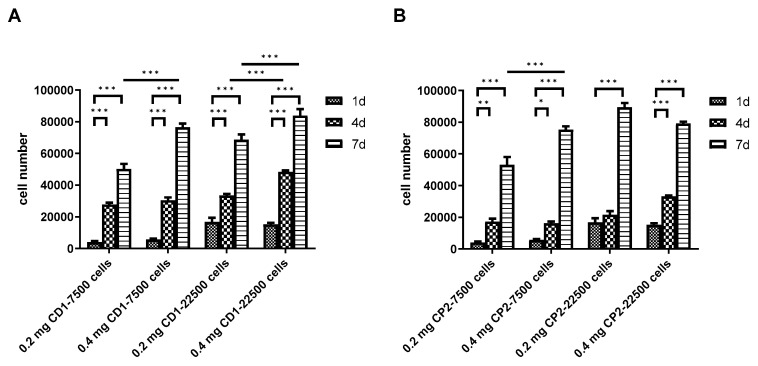
Changes in the number of viable DPSCs cultivated with (**A**) Cytodex 1 (CD1) or (**B**) Cytopore 2 (CP2) in 96-well non-attaching plates under static conditions for 7 days. Initially 7500 and 22,500 cells were seeded into the wells containing 0.2 or 0.4 mg/mL microcarrier. Values are means ± SEM (*n* = 5). * *p* < 0.05, ** *p* < 0.005, *** *p* < 0.001 in comparisons as indicated.

When DPSC viability on the microcarriers was examined using the alamarBlue assay, on the first day after seeding, viability was independent of the microcarrier concentration used and depended only on the initial cell number. Four days after seeding, cell numbers significantly increased in all groups compared to day one in both the Cytodex 1 and Cytopore 2 microcarriers (Figure 8A,B). By day 7, cell numbers further increased. During the Cytodex 1 application a greater number of microcarriers led to a significantly higher final DPSC number (Figure 4a). Additionally, a higher initial cell number (22,500) combined with the Cytodex 1 resulted in higher viable cell counts than the lower initial cell number (7500) (Figure 8A). In the case of the Cytopore 2, at day 7 a higher number of microcarriers did not result in a significantly higher final DPSC number. On the other hand, cultures with the higher initial cell number (22,500) also showed higher viable cell counts than cultures with the lower initial cell number (7500), independent of the microcarrier concentration (Figure 8B). 

### 3.6. Scaling-up Experiments under Dynamic Conditions

**Figure 9:**
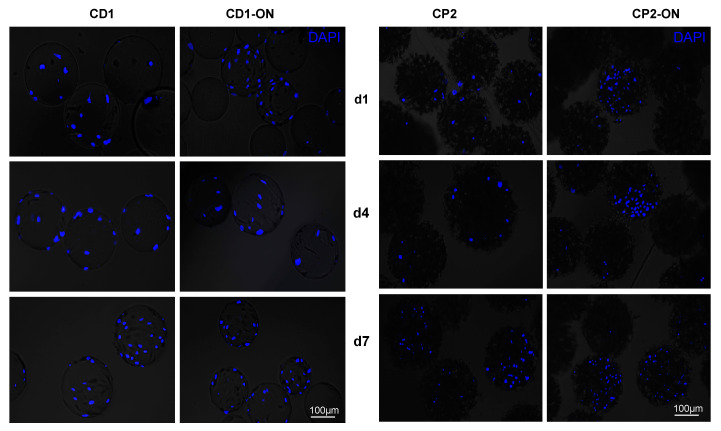
Attachment of DPSCs to the microcarriers after 1, 4 and 7 days of incubation in shake flasks, as visualized by fluorescence microscopy after DAPI staining. Half of the flasks (CD1 and CP2) were immediately shaken in an incubator. The other half of the flasks (CD1-ON and CP2-ON) were left still in the incubator overnight before shaking started, to promote cell adhesion to the microcarriers. The scale bar indicates 100 µm.

The cells on Cytodex 1, and especially on Cytopore 2, were hardly visible by phase contrast microscopy. To make the cells growing on the surface of the microcarriers visible, samples were stained with DAPI dye, and the fluorescent nuclei were photographed with a fluorescence microscope. Images of the cultures are shown in Figure 9. It was interesting to notice that on Cytodex 1 microcarriers, cell distribution on the microcarrier surfaces was mostly random, independent of whether the cultures were placed on the shaker immediately after seeding or incubated overnight before shaking started (Figure 9, left panel). On the Cytopore 2, uneven distribution was seen as both strongly overgrown and almost empty spots on the microcarriers. Just as in the Cytodex 1, in the case of the Cytopore 2 microcarrier, DAPI staining demonstrated that cells grew on the surface of the microcarriers during shake flask culture (Figure 9, right panel). An uneven cell distribution could be observed in Cytopore 2 cultures in the samples taken on days 1, 4 and 7 when the flasks were shaken only after standing still overnight. In the Cytopore 2 group, when the cells and microcarriers were shaken immediately after seeding, the number of cells on the microcarrier was considerably lower than in the other groups and their distribution was random (Figure 9, right panel).

**Figure 10:**
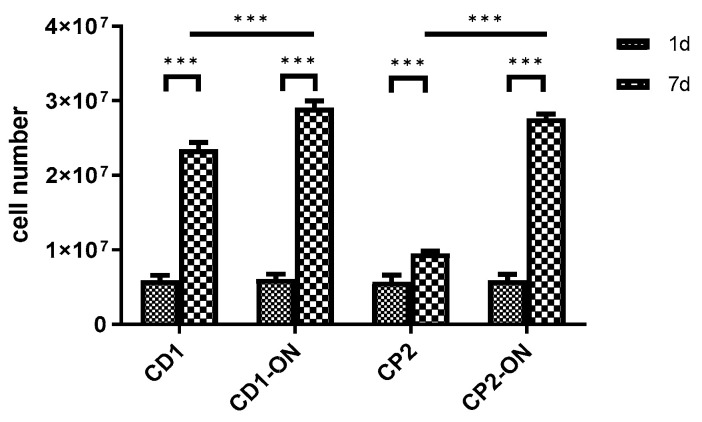
Changes in the number of viable DPSCs cultivated with Cytodex 1 (CD1) or Cytopore 2 (CP2) under dynamic cultivation in shake flasks for 7 days. Half of the flasks (CD1 and CP2) were immediately shaken in an incubator. The other half of the flasks (CD1-ON and CP2-ON) were left still in the incubator overnight before shaking started, to promote cell adhesion to the microcarriers. Values are means ± SEM (*n* = 5). *** *p* < 0.001 in comparisons as indicated.

Cell viability assays were performed on days 1 and 7 of cultivation. At day 1, the alamarBlue assay showed that cell numbers were similar in all four groups independent of the type of microcarrier and whether culture shaking started immediately or only after a static overnight incubation. On the seventh day of the experiment a considerable increase in the number of DPSCs was observed in all groups yielding 10–30 million DPSCs in each flask. The results also demonstrated a significantly higher number of living cells after a 12 h static incubation on both Cytodex 1 and Cytopore 2 compared to those immediately shaken without an initial static incubation (Figure 10). This difference was more pronounced for cells cultured on Cytopore 2 microcarriers, on which the number of DPSCs only increased slightly by day 7 if the flasks were immediately shaken after cell seeding (Figure 10). These data suggest that the DPSCs were less adherent to the Cytopore 2 microcarrier when shaken from the beginning. 

### 3.7. Glucose Consumption by Cultured Cells

**Figure 11:**
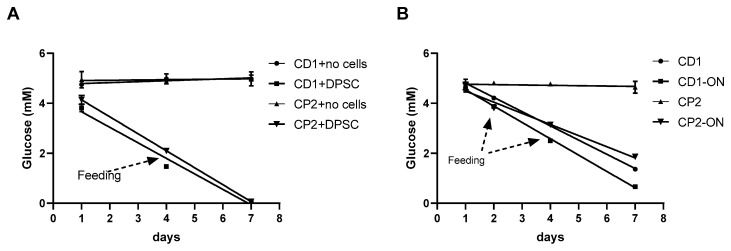
Glucose utilization by DPSCs cultivated with Cytodex 1 (CD1) or Cytopore 2 (CP2) in 96-well plates under static conditions and in shake flasks under dynamic conditions for 7 days. (**A**) Static conditions in 96-well plates. Microcarriers were cultured in static conditions without DPSCs (CD1, CP2) or with DPSCs (CD1+DPSC, CP2+DPSC). The medium was partially changed at day 4. (**B**) Dynamic cultivation in shake flasks. Flasks were either immediately shaken (CD1, CP2 or were left) in the incubator overnight before shaking started (CD1-ON and CP2-ON). The medium was partially supplemented at day 2 and then partially changed at day 4. Values are means ± SEM (*n* = 5).

To investigate glucose consumption, the DPSCs were first cultured under static conditions using the same microcarrier concentrations as described above. As expected, no glucose consumption was observed when the microspheres were incubated in a cell-free medium (Figure 11A), but, when the DPSCs were added to the microcarriers, the cells utilized all available glucose in the culture medium independent of whether they were incubated on Cytodex 1 or Cytopore 2 (Figure 11A). 

When dynamic culture conditions were applied in shake flasks, we observed a progressive utilization of glucose in three of the four groups. Glucose consumption in both Cytodex 1 groups, and in the Cytopore 2 group which was not initially shaken overnight, showed very similar trends, leading to the actual exhaustion of this energy source in the medium by day 7, despite the batch-feeding of flasks at days 1 and 4 with fresh medium (Figure 11B). In contrast, glucose consumption in the Cytopore 2 group given immediate initial shaking was low, as the number of living cells was also low under these conditions. (Figure 11B).

## 4. Discussion

In the present proof-of-concept study we compared the suitability of two microcarriers for culturing ectomesenchymal DPSCs in static and dynamic conditions. We found that both non-porous Cytodex 1 and porous Cytopore 2 provided appropriate conditions for scaling up dental pulp cells that included stem cells. Thus, the tested expansion technologies can provide a safe and effective way to supply DPSCs for diagnostic and therapeutic applications.

As expected, human DPSCs adhered to surfaces following seeding and started to divide, showing a doubling time of approximately two days under stationary conditions. In this culture condition we found that the vast majority of cultured cells were fibroblast-like, having a spindle-shaped structure. Our analyses of the DPSCs prior to expansion is similar to previous reports [8,46,47,48,49], showing that the cells are not homogeneous for Stro-1, which is generally regarded as a key stem cell surface marker, with some cells immunostaining for nestin and vimentin [11,12]. Our findings are in line with many other observations [8,46,47,48,49] indicating that the proportion of STRO-1-positive cells ranged between 10% and 20%. Dental pulp stem cells, as well as partially differentiated dental pulp cells, continue to differentiate through subsequent passages. Therefore, the dental pulp cell population, which contains a significant portion of stem cells, must be scaled up without continuous passaging. 

As found in previous studies, the cells doubled in approximately two days [3,11,12,45,48], and likely further decreased the relative number of stem cells in culture. When cells are seeded at optimal concentrations without microcarriers, an approximately 10-fold increase in viable cells can be achieved, i.e., 10^6^ cells after seven days. Afterwards, cell numbers reach a plateau and further expansion can only be achieved by passaging the cells. Therefore, cell production during small-scale, static cultivation of DPSCs yields only very limited numbers of cells for tissue engineering and other applications. 

Microcarriers are small, spherical beads developed for production of cultured cells at a high density. Dynamic systems such as shake flasks, stirring flasks and bioreactors maintain the microcarriers in suspension [50]. Under these conditions anchorage-dependent cells, such as mesenchymal stem cells, cannot survive and proliferate for long without a firm surface that provides attachment for them [51]. As mesenchymal stem cell growth is anchorage-dependent, and their interactions with the microcarrier surface is critical. Additionally, microcarrier surfaces also adsorb proteins and other components of the culture medium to further facilitate cell attachment [51,52]. Of the extremely wide range of available microcarriers, we selected two prototypes, non-porous Cytodex 1 and porous Cytodex 2, for characterizing quantitatively the scaling-up process for the ectomesenchymal DPSCs. These microcarriers have already been demonstrated to be very useful for expanding cells of various origins including mesenchymal stem cells [30,53,54]. 

In our study, conventional phase contrast microscopy clearly showed that elongated spindle-shaped DPSCs effectively adhered to Cytodex 1 microcarriers. Because of its rough surface, the cells are not visible on the surface of Cytopore 2 by phase contrast microscopy; however, the application of DAPI staining and fluorescent microscopy, and also Vybrant DiD staining followed by two-photon microscopy, revealed that DPSCs were bound to the surface of both Cytodex 1 and Cytopore 2 under static culture conditions. While the cells bound to the Cytodex 1 randomly, their distribution on the Cytopore 2 was not homogenous, probably because of the local disunity of the porous microcarrier. In the cell viability experiments we used two different cell concentrations and two different microcarrier densities to find optimal conditions for such arrangements, based on our preliminary experiments and on literature data. These studies both showed a considerable increase in living cell numbers four days after seeding, which was further increased by day 7. Each initial DPSC concentration and each applied Cytodex 1 or Cytopore 2 carrier number resulted in a significant and reasonable increase in cell number. The 5- to 10-fold expansion of viable cells on microspheres under stationary conditions was quite comparable to the 10-fold multiplication of cells in cultures without microspheres, but the great advantage of the use of microcarriers is the possibility of increasing culture volume in three dimensions without the passaging that is required when cells are grown without microspheres in two dimensions under static conditions. 

Cytodex 1 and Cytopore 2 are widely used for cell expansion studies of mesoderm-derived attaching cells but ours is the first quantitative study to investigate the scaling-up capabilities of these microcarriers for mesenchymal cells derived from the mesoderm. Nevertheless, our data are in line with recent studies showing that Cytodex 1 is an efficient expansion microcarrier for stem cells of adult umbilical cord [55] early [56] and adult [57] bone marrow, as well as MSCs of porcine origin [41]. Cytodex 2 also serves well as an upscaling microcarrier for MSCs [41], for chondrocytes [58] and for fibroblasts [59]. Of the studies which tested the detachability of MSCs and other attaching cells, all reported that detachment was very difficult and that most cells remained attached to the microcarrier or died during the isolation procedure [41,55,56,57]. 

Although there are many studies describing the cultivation of mesenchymal stem cells on microcarriers [30,53], only two studies report DPSCs cultivated on microspheres under static, non-shaking conditions [38,60]. Their results are in line with our observations. Bhuptani and Patravale cultured DPSCs with and without PGLA microcarriers in static conditions using 96-well plates. The MTT assay which is directly proportional to cell viability increased 10-fold between culture days 1 and 7. Their results showed that the PLGA microscaffolds did not diminish the viability of stem cells [38]. Zhang and coinvestigators studied the growth of DPSCs on Cytodex 3 using 24-well plates, also under stationary conditions. Cell proliferation was assessed with a CCK-8 cell viability assay. DPSCs attached to Cytodex 3 microcarriers showed a fibroblast-like morphology. Cell numbers increased 1.5-fold during the 3-day cultivation of cells on Cytodex 3. When a 7-day incubation was performed, no absolute numbers were given, only various treatment schedules were compared and fold differences between them provided [60]. Neither of these studies explored the possibility of using dynamic culture conditions

When we cultured cells in shake flasks with Cytodex 1 and Cytopore 2, cell attachment was also observed; however, attachment increased in both groups with an initial overnight incubation of the flasks without shaking. This observation indicates that, after seeding, DPSCs need time to attach to the microcarriers, otherwise their adherence to the surface of microcarriers is diminished. Both the morphological analysis and the cell viability assays by alamarBlue tests supported this conclusion. Additionally, the outcome of our dynamic experiments in shaking flasks is encouraging as DPSCs could be greatly scaled up to produce 3 × 10^7^ cells per flask. This level of cell numbers is comparable to those which are used in cell therapy applications [30,53]. Mesenchymal stem cell cultivation on microcarriers has been widely characterized [30,53]. 

To date, only three studies have reported DPSC cultivation on microspheres in dynamic, three-dimensional shaking conditions [38,40,60,61,62]. These three papers used dynamic conditions but were concerned only with cell differentiation and gene expressional changes and lacked any quantitative cell viability or cell proliferation data [40,61,62]. Cell expansion of DPSCs on microcarriers has been characterized in only two studies, using stationary and not dynamic conditions, as described above [38,60]. 

The continuous supply of energy is an important but often neglected problem during stem cell scaling up. Therefore, we addressed this in our proof-of concept study. When the glucose consumption of the DPSCs on Cytodex 1 or Cytopore 2 microcarriers was investigated under static conditions, cells exhausted all available glucose in the culture medium in 7 days. Likewise, as seen in other studies [29,30,31], we found that when a dynamic shake flask culture was used the progressive utilization of glucose had depleted this energy source by day 7, in spite of the partial refeeding of the flasks with fresh medium at days 1 and 4. Therefore, the replenishment of glucose, to provide optimal glucose concentration during extended culture periods, is necessary for a high yield of stem cells [29,30,31].

Like other cells, DPSCs bind strongly to both Cytodex 1 and Cytopore 2 microcarriers and therefore cannot be detached with standard methods for cell release using enzymes such as trypsin. For this reason, it was not possible to perform further cell characterization studies, after expansion, for comparison with those done prior to the scaling-up procedure. Furthermore, trypsinization can cause cell damage, and therefore in the future we will explore other possibilities such as the application of ultrasonic traveling waves [63]. Nevertheless, the present proof-of-concept work represents an important step paving the way for future research to characterize not only the growth dynamics but also the functional characteristics of DPSCs after expansion. 

In parallel research we work on the development of scaffolds that allow not only the survival and proliferation of the ectomesenchyme-derived cells, both in vitro and in vivo, but also elicit their differentiation [64,65,66,67,68]. By chemical modification of biocompatible and biodegradable poly(aspartamide) (PASP)-based hydrogels, and parallel studies on their biocompatibility, biodegradability and regenerative potential, we are attempting to develop novel scaffolds that are clinically applicable [64,65,66,67,68]. We expect that in the near future these separate lines of research, on microcarriers and scaffolds, will meet through the development of biomaterials that can serve both for cell expansion and tissue regeneration.

Our work clearly shows the possibility of large-scale expansion of dental pulp cells under dynamic conditions. This will, for the first time, make it possible to grow enough cells at low passage to allow the isolation of the unique populations of stem cells contained within the dental pulp. DPSCs originate from the cranial neural crest ectomesenchyme, i.e., from the ectoderm and not the mesoderm (Yoshide 2020, Mead 2016). Here we have demonstrated for the first time the successful quantitative expansion of the ectomesenchymal cells, i.e., the DPSCs, on microspheres. Recent pioneering studies have clearly demonstrated that MSCs of different origins exhibit very different proliferation and differentiation potential and also interact with various scaffolds [34,35,36,37]. Therefore, our present work is important in providing evidence about the capability of DPSCs to grow on cell attaching microcarriers under dynamic conditions. Moreover, stem cell populations within the pulp may make the dental pulp a robust source of autologous stem cells for healing and tissue regeneration after expansion. 

## 5. Conclusions

There is an urgent need to develop effective, reproducible and safe scaling-up methods producing large amounts of therapeutically active, dentally derived stem cells. Microcarrier-based culturing in dynamic, three-dimensional conditions is a promising technology combining a required large growth surface area with controlled conditions. In the present proof-of-concept study we have demonstrated the suitability of both non-porous and porous microcarriers to culture ectomesenchymal DPSCs in static and dynamic conditions. The tested scaling-up method may help to develop a good DPSC supply technology for diagnostic, and therapeutic applications, including strategies for co-culture with ameloblast lineage cells for tooth regeneration.

## Data Availability

The data supporting the conclusions of this article will be made available by the authors, without undue reservation.

## Supplementary Materials

The following videos are available online at https://www.mdpi.com/article/10.3390/polym13223951/s1, Figure S1: DPSC cells on CD1_2photon microscopy_VybrantDID staining and Figure S2: DPSC cells on CP2_2photon microscopy_VybrantDID staining.
